# A Further Protest against the Routine Use of Purgatives

**Published:** 1912-02

**Authors:** 


A Further Protest Against the Routine Use of Purgatives. Edwin Walker of Evansville, Ind. (*Am. Jour. Obst.* November, 1911), renews his advice against the promiscuous use of laxative remedies. The popular faith in these drugs is akin to a superstition, and for this the physician is largely responsible. In the past seven years he has not purged 1 per cent. of his operative cases. His patients have done better, they have had fewer complications, and have been more comfortable. More than 90 per cent. of his cases have been addicted to the purgative habit. Ninety-five per cent. of these were relieved of their constipation under a suitable diet combined with regular habits. The routine practice of using a purge before every operation is bad. The bowel contents are rendered more fluid and germ activity is increased.

